# Supplementary material for: Synthetic microbe communities provide internal reference standards for metagenome sequencing and analysis
Source: Nat Commun. 2018 Aug 6;9:3096. doi: 10.1038/s41467-018-05555-0 (PMC6078961; doi:10.1038/s41467-018-05555-0)
Supplement: Supplementary file 1 — Supplementary Information [file 41467_2018_5555_MOESM1_ESM.pdf]

SUPPLEMENTARY MATERIALS

Synthetic microbe communities provide internal reference standards for metagenome sequencing and analysis

Hardwick *et al.*

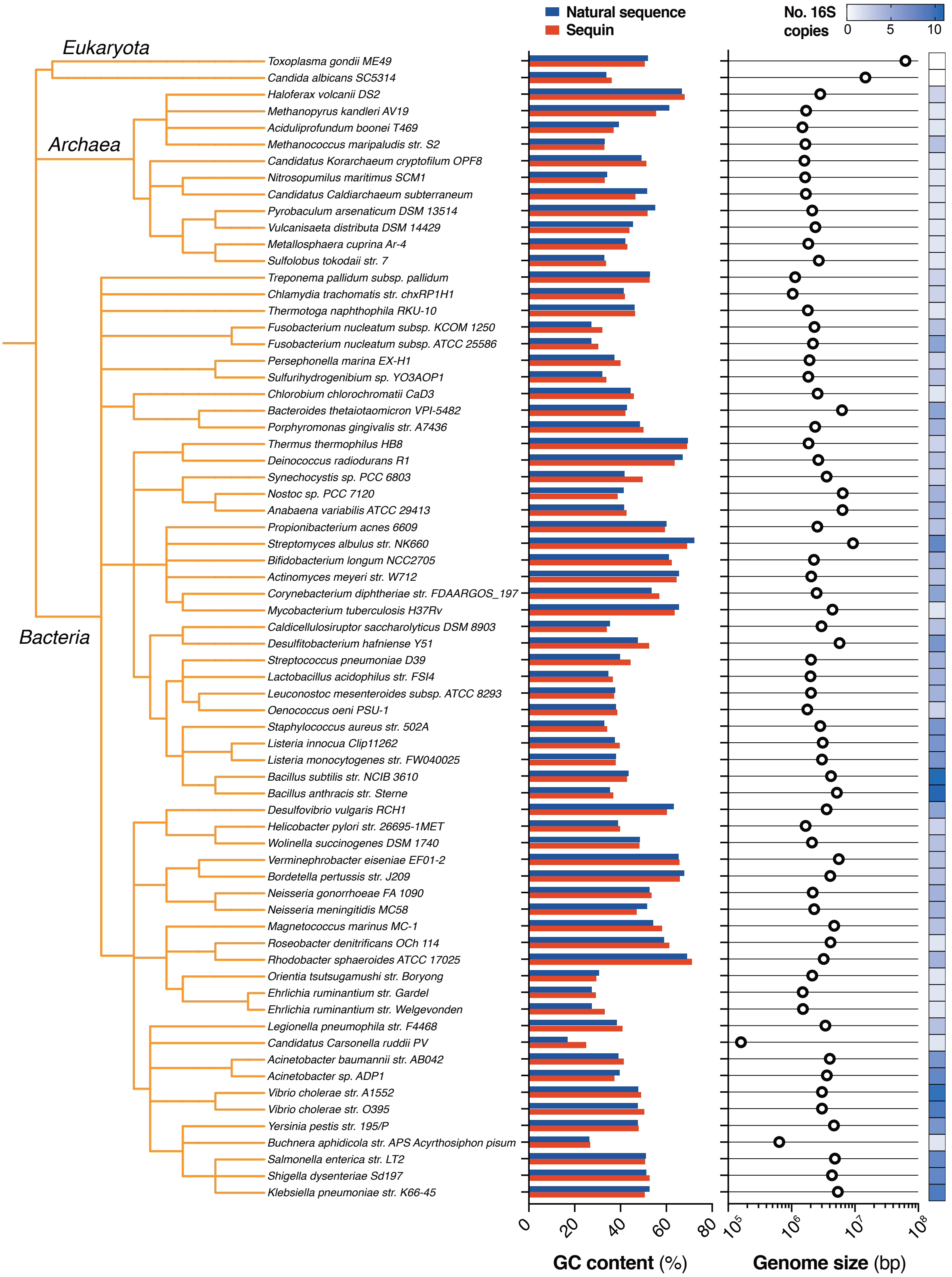

Supplementary Fig. 1: Artificial genomes mirror the complexity of a natural microbial community. To design artificial sequences, we sampled a diverse selection of finished microbial genomes that encompassed a wide representation of taxa (including Eukaryota, Bacteria and Archaea), size (0.5-10 Mb for prokaryotes), GC content (~20-70%), rRNA operon count (1-11) and isolation from a diverse range of environments (human body, aquatic, terrestrial and extreme physical or chemical conditions). Phylogenetic tree shows the evolutionary relationships of the 69 microbes that were chosen. The GC content of each sequin closely matches that of the microbial genome from which it was derived.

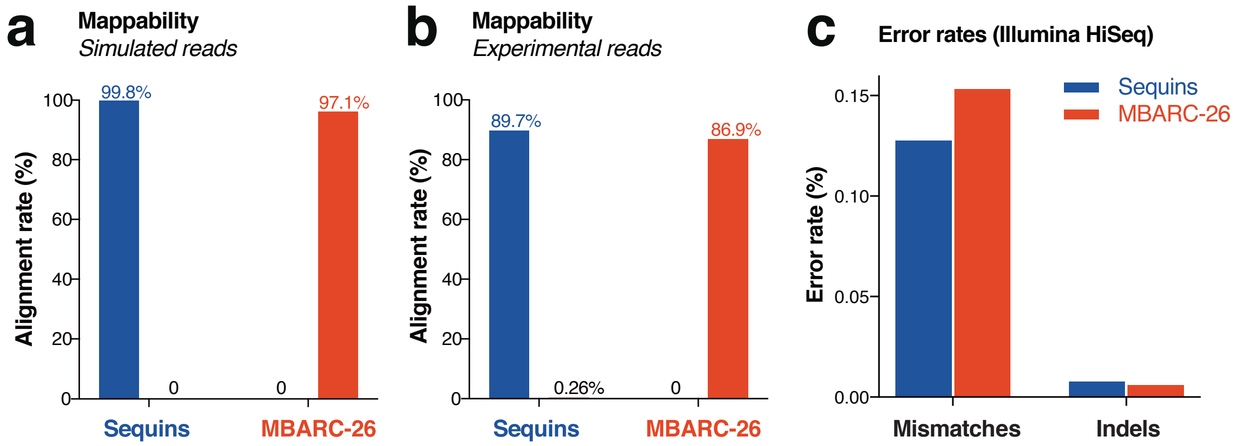

Supplementary Fig. 2: Comparison of synthetic DNA standards to mock microbial community. To demonstrate that sequins perform comparably to natural microbial genomes, we compared sequins to a mock microbial community comprising 23 bacterial and 3 archaeal genomes ('MBARC-26'). By mapping reads to a combined genome index comprising the sequins (blue) and MBARC-26 genomes (red), we observed comparable mappability of sequins and MBARC-26 genomes, first using simulated read libraries **(a)** and then experimental reads **(b)**. With simulated reads, there was no cross-alignment of reads; i.e. no sequin-derived reads mapped to any MBARC-26 genome, and vice-versa. With experimental reads, a negligible fraction of reads (0.26%) from our neat sequins library mapped to MBARC-26 genomes, which likely represent a low but detectable level of microbial contamination in laboratory reagents. **(c)** After spiking MBARC-26 gDNA with sequins, we observed comparable sequencing error rates (mismatches and indels) for sequins and MBARC-26 genomes.

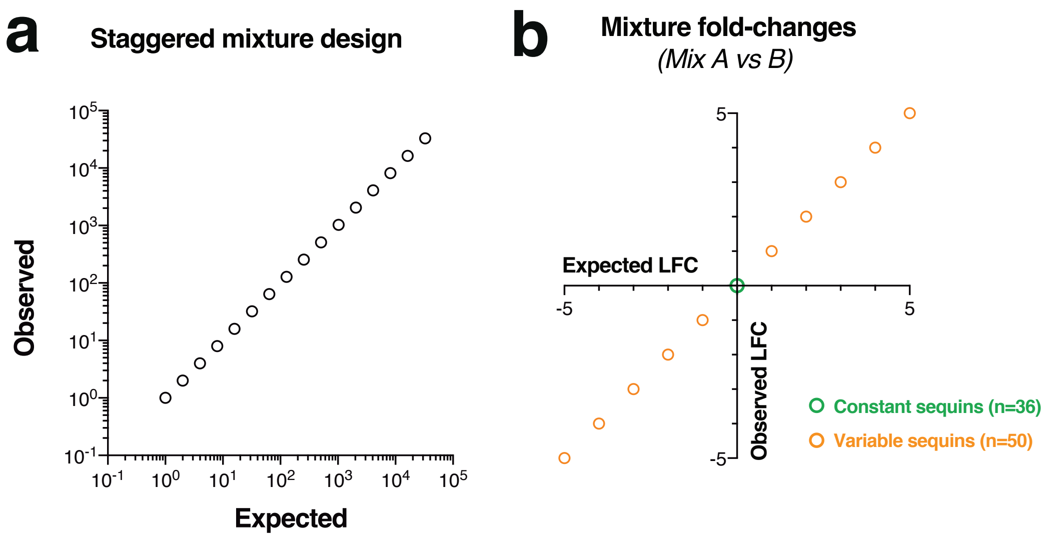

Supplementary Fig. 3: Mixture design for metagenome sequins. (a) After synthesizing each individual DNA standard separately, we combined them into a staggered mixture ('Mix A') which spans a ~3.2 x 10^4-fold concentration range (designed to mimic that of a natural metagenome sample). The mixture was designed with 16 staggered concentration points, with at least 5 standards per point (comprising a range of lengths and GC%). **(b)** We then prepared an alternative mixture ('Mix B') containing the same set of 86 sequins, but with a subset that undergo known $\log_2$ fold-changes (LFC) between mixtures (green; n=50) and a subset that remain at equimolar concentrations (yellow; n=36). This design allows for measurement of fold-changes between samples, while also providing negative controls for use in normalization.

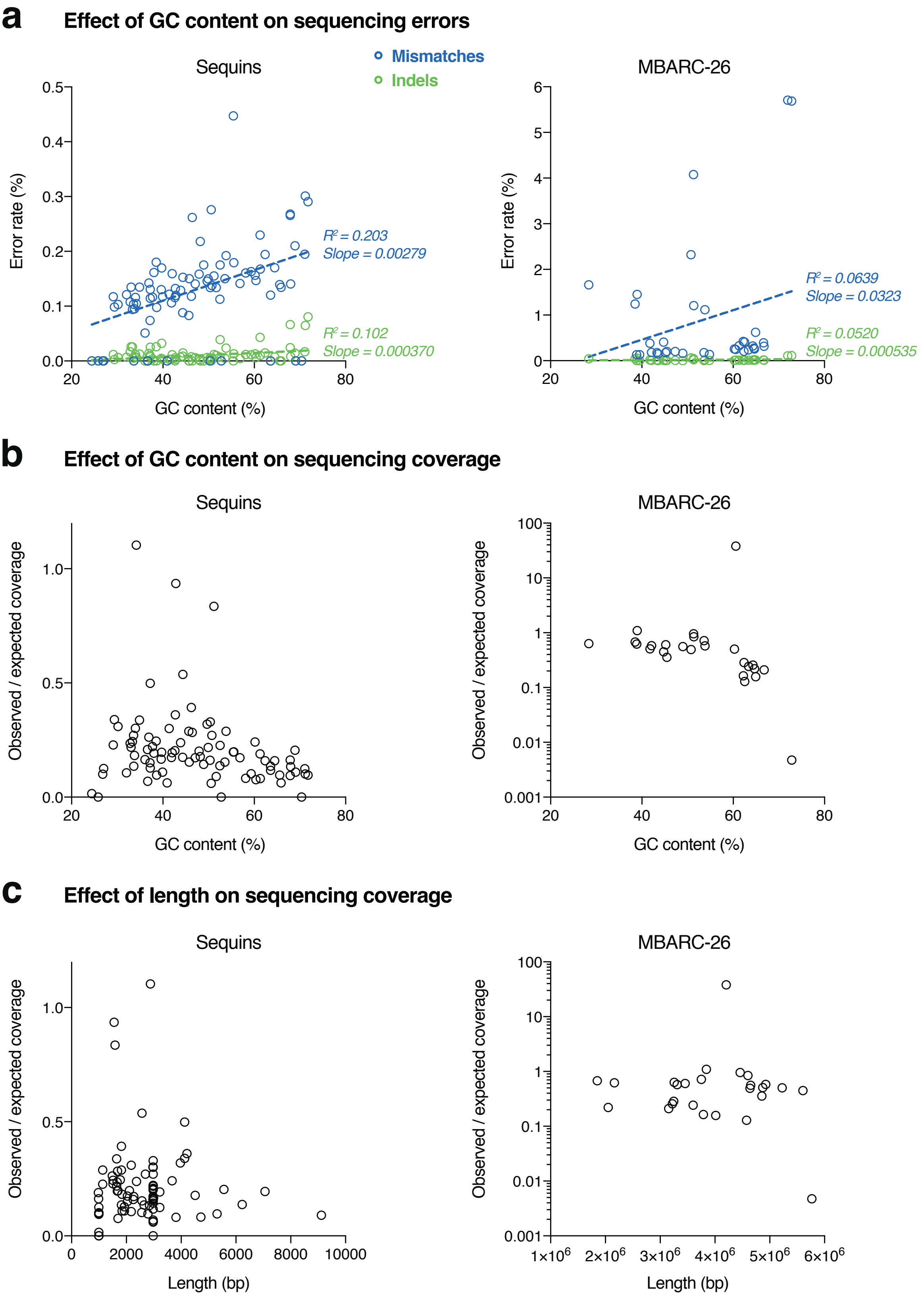

Supplementary Fig. 4: Using sequins to assess sequencing coverage biases. (a) Scatter plots show rate of mismatch (blue) and indel (green) errors plotted against respective GC content. Dashed lines represent linear regression models fitted to the data. Sequins are shown on the left, MBARC-26 genomes on the right. **(b)** Scatter plots show the fold-coverage of each sequin (observed divided by expected) plotted against respective GC content. **(c)** Scatter plots show observed/expected fold-coverage plotted against respective length.

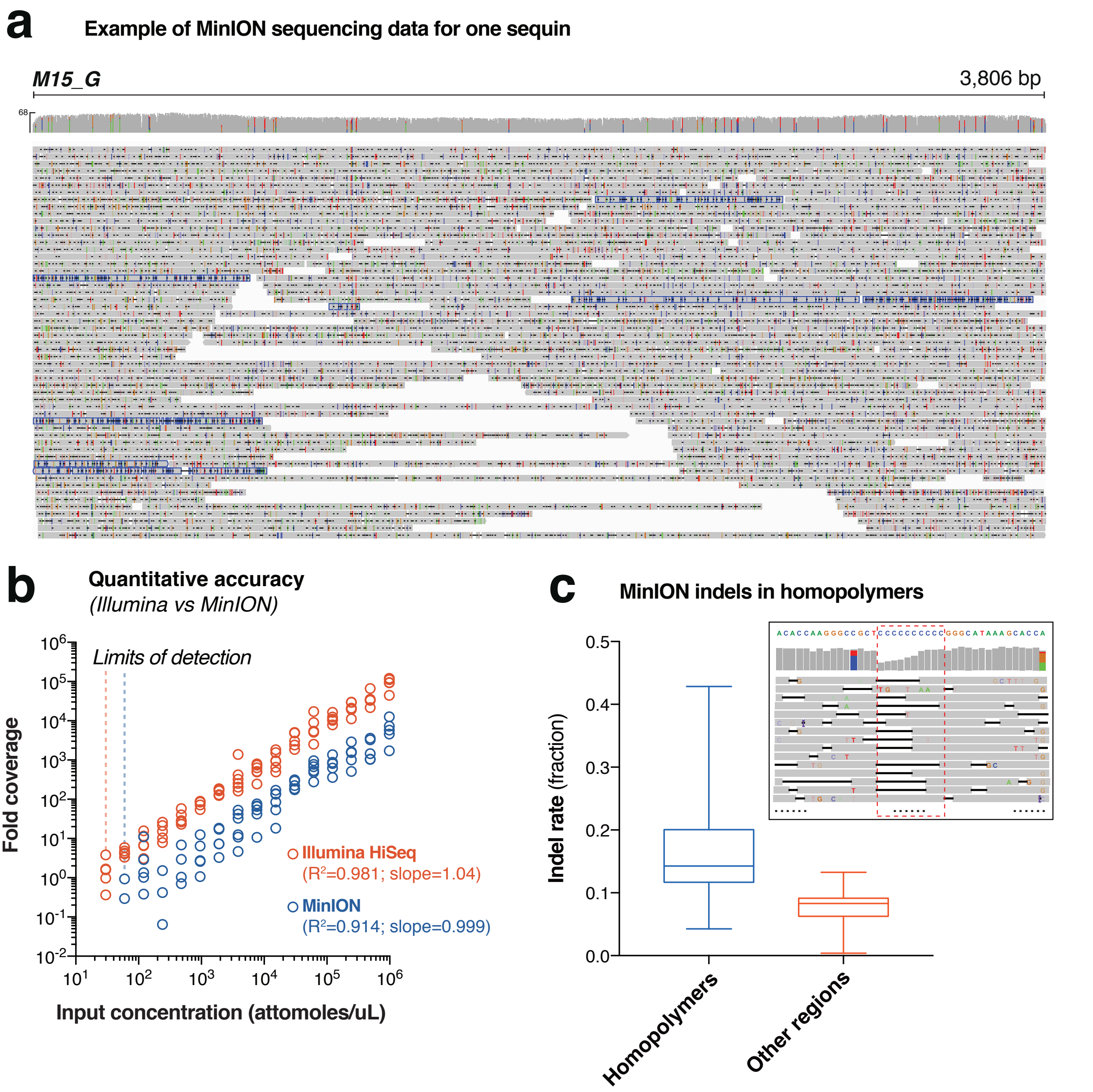

Supplementary Fig. 5: Long-read sequencing with the MinION platform. (a) Most sequins (61 out of 86) were fully covered by at least one single MinION read. Genome browser view shows example of one sequin (*M15_G)*. **(b)** Scatter plot shows the quantitative accuracy of sequencing the neat sequins mixture on the Illumina HiSeq (red) compared to Oxford Nanopore Technology's MinION platform (blue). The limits of detection for the two instruments are indicated in vertical dashed lines. **(c)** Box plots show that, using sequins, MinION sequencing has significantly higher per-base indel rates in homopolymeric sequences (blue) compared to all other regions (red). Box center lines indicate median; bounds of boxes indicate upper and lower quartiles; whiskers extend to min/max values. Inset box shows genome browser view of characteristic MinION indels in a homopolymeric sequence located in *GC_74_1* (red dashed box). Note the dramatic sequence coverage drop-off at the upstream end of the homopolymer tract.

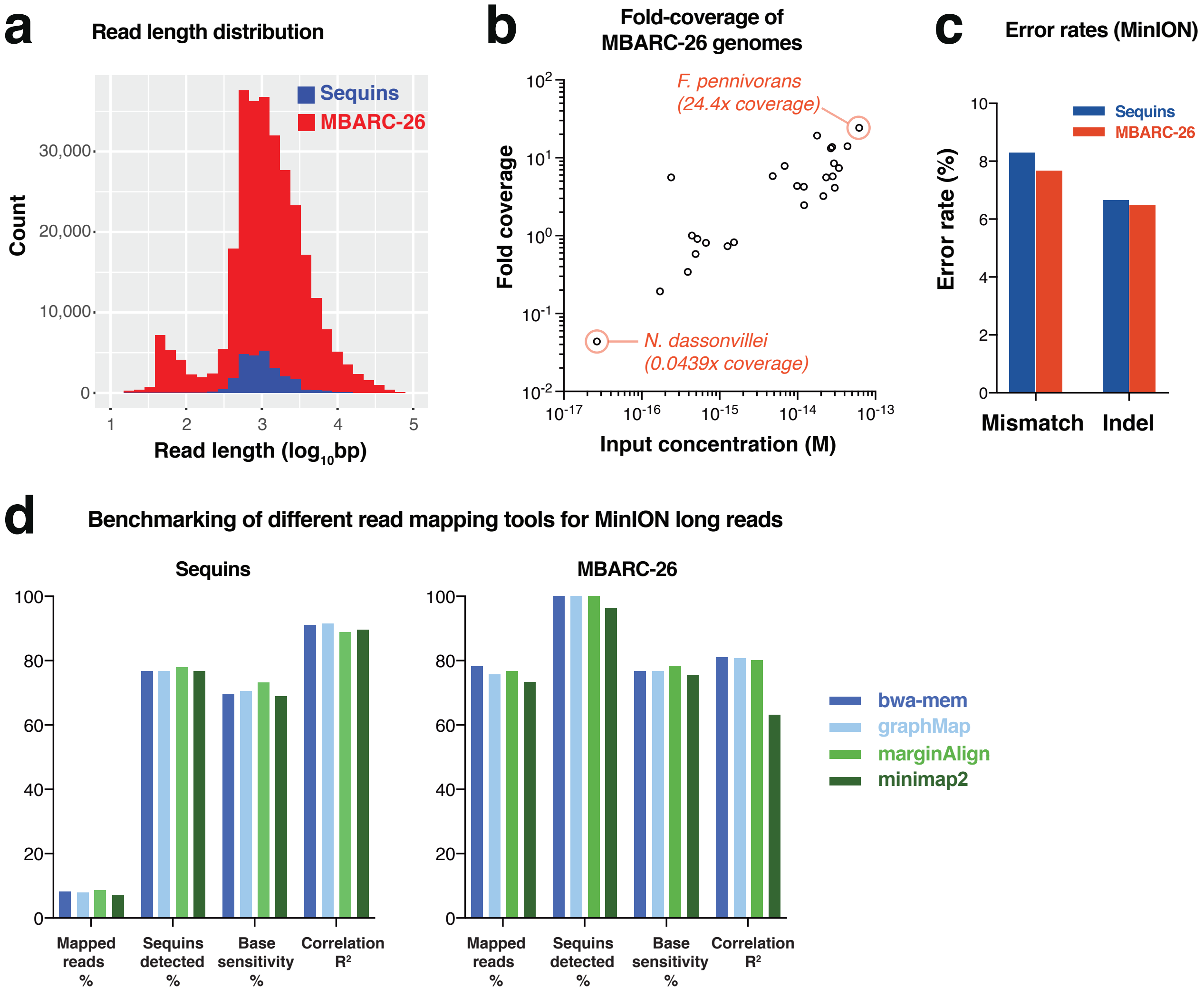

Supplementary Fig. 6: Using sequins to benchmark MinION sequencing and analysis. We spiked MBARC-26 community gDNA with sequins (5% fractional abundance), and sequenced the combined sample on the MinION instrument. **(a)** Frequency histograms show the read length distribution for reads aligning to sequins (blue) and MBARC-26 genomes (red). We observed several reads >100 kb which aligned to MBARC-26 genomes. **(b)** Scatter plot shows the average fold-coverage of each MBARC-26 genome, plotted against its respective molarity in the mixture. Fold-coverage varied from as low as 0.0439x for *Nocardiopsis dassonvillei* up to 24.4x for *Fervidobacterium pennivorans*. **(c)** Bar charts show the sequencing error rates (mismatch, left; indel, right) for sequins (blue) and MBARC-26 genomes (red). **(d)** We used the spiked MBARC-26 library to benchmark the performance of a range of different read-mapping tools designed for long reads (bwa-mem, graphMap, marginAlign and minimap2). Performance metrics for sequins are shown on the left, while MBARC-26 genomes are shown on the right.

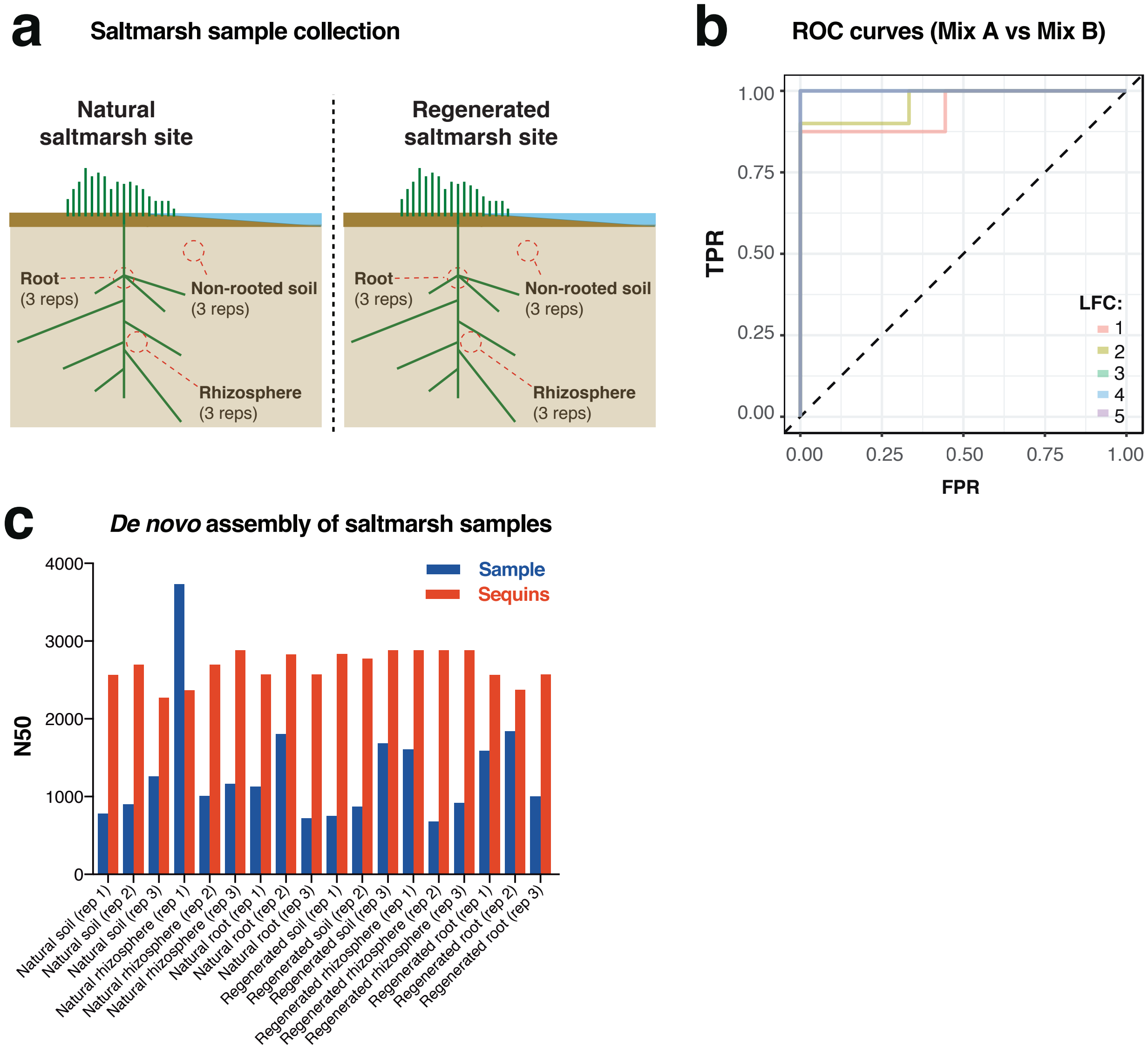

Supplementary Fig. 7: Using sequins to profile saltmarsh metagenomes of unknown content. (a) We sought to compare the microbe composition of three types of samples (non-rooted soil, rhizosphere and root) collected from two different environments (natural, regenerated). Three replicates of each sample type were collected from each site, for a total of 18 samples. The ‘natural’ site (left) represented a saltmarsh environment undisturbed by human development, while the ‘regenerated’ site was re-established as part of the Sydney 2000 Olympics after being previously disturbed by human development for decades. Sequins Mix A and B were alternately spiked into natural and regenerated samples, respectively. **(b)** Receiver operator characteristic (ROC) curves show the diagnostic performance of sequins between Mix A and Mix B. Sequins are colored by their respective $\log_2$ fold-change (LFC) groups. TPR: true-positive rate; FPR: false-positive rate. **(c)** Bar graphs show N50 values for contigs aligned to sequins (red) and contigs not aligned to sequins (blue).

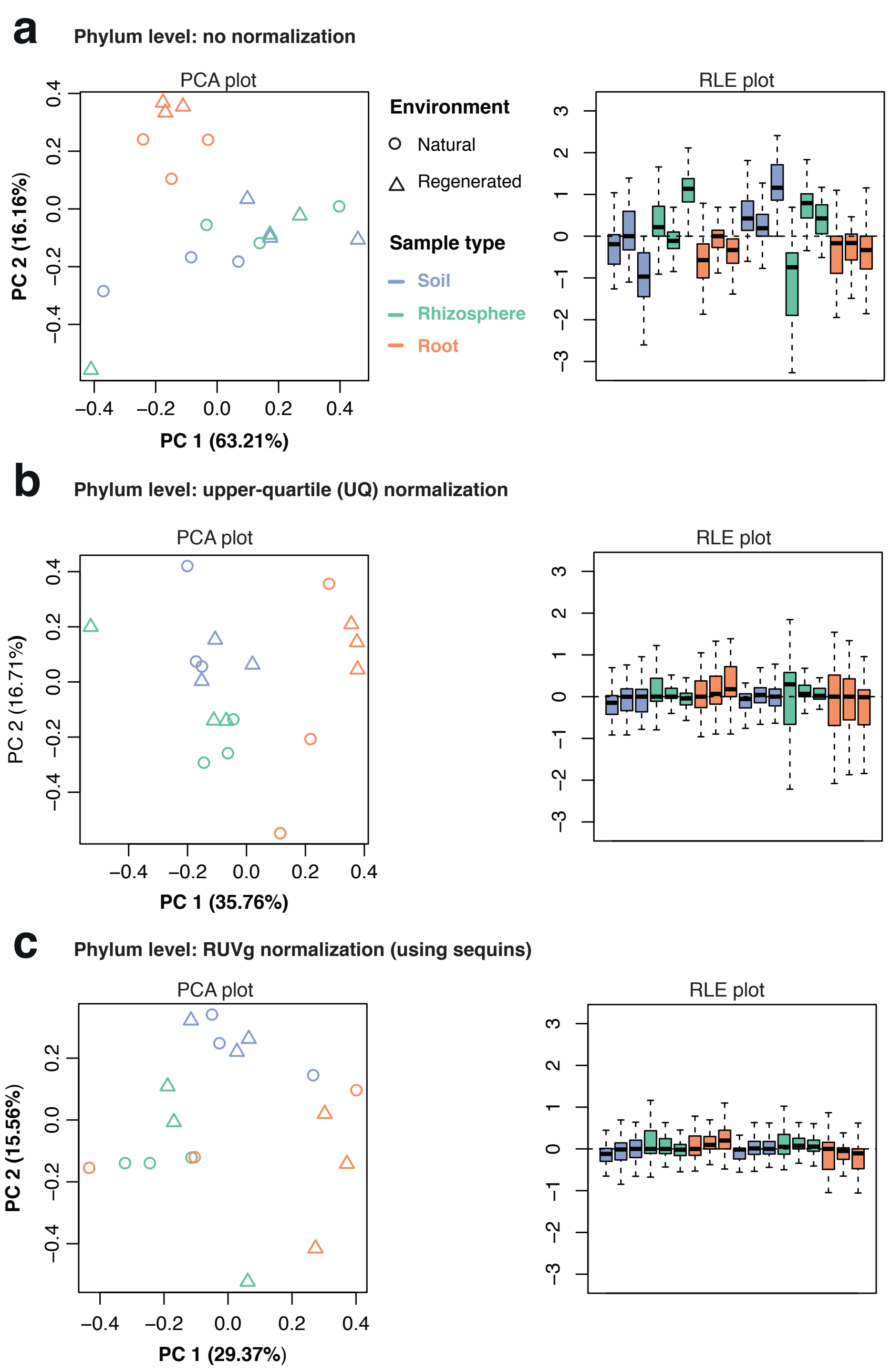

Supplementary Fig. 8: Normalization results for saltmarsh samples (phylum level). (a) Principal component analysis (PCA) results (left) based on taxonomic classification of samples against the RefSeq database (phylum level; n=75 phyla), without any normalization. Samples clustered by sample type (soil, blue; rhizosphere, green; root, red) rather than environment (natural, circles; regenerated, triangles). Relative log expression (RLE) plots (right) demonstrate a clear need for normalization, with samples not centered around zero and most samples showing high variability. **(b)** PCA results (left) and RLE plots (right) after upper-quartile (UQ) normalization of the data. RLE plots show some improvement, with samples mostly centered around zero. However, some samples still displayed excessive variability, e.g. replicate 1 of the rhizosphere sample taken from the regenerated site. **(c)** PCA results (left) and RLE plots (right) after RUVg normalization of the data. The RUVg method uses the subset of sequins that remain at a fixed concentration between mixtures as negative controls to adjust for unwanted variation. RLE plots demonstrate further improvement following RUVg normalization, with all samples centered around zero and the excessive variation of most samples now removed.

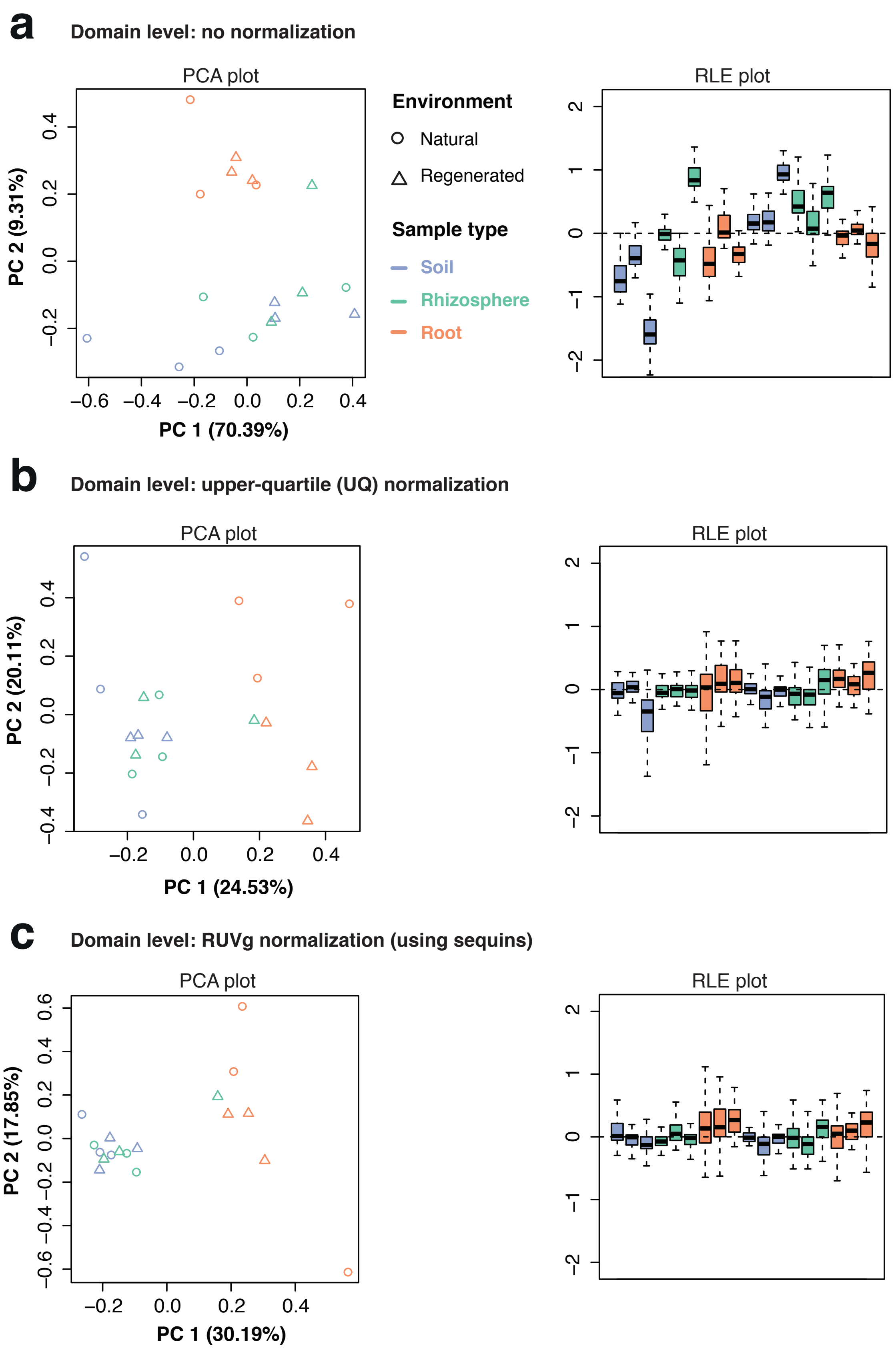

Supplementary Fig. 9: Normalization results for saltmarsh samples (domain level). Normalization results after taxonomic classification of samples against the RefSeq database (domain level; n=3 domains). (See Supplementary Fig. 8 legend for further details).

	Illumina	Oxford Nanopore
**Instrument**	HiSeq 2500	MinION
**Library prep.**	Nextera XT Sample Prep Kit	MinKNOW v1.7.3
**Input DNA**	1 ng	1 µg
**No. reads**	17,681,564 (pairs)	160,709
**Aligner used**	Bowtie2 (default)	bwa-mem (–x ont2d)
**No. mapped reads (%)**	16,182,491 (91.5%)	124,239 (74.0%)
**No. sequins detected (%)**	86 (100%)	75 (87.2%)
**Base-level sensitivity** [a]	96.8%	80.0%
**Quantitative accuracy** [b]	0.981	0.914
**Depth of coverage (mean)**	13,999x	724x
**Mismatch error rate (%)**	0.127	7.12
**Indel error rate (%)**	0.00770	8.71

Supplementary Table 1: Comparison of Illumina and Oxford Nanopore library statistics.
Metrics and parameters describing the sequencing of a neat preparation (i.e. no natural DNA added) of metagenome sequins (Mix A).
[a] Base-level sensitivity is defined as the fraction of reference bases covered by aligned reads.
[b] Quantitative accuracy is defined as the correlation coefficient (R^2) obtained after fitting a linear regression model to observed coverage plotted against input concentration.

	Metagenome sequins				MBARC-26 genomes			
	**Bwa-mem**	**GraphMap**	**marginAlign**	**minimap2**	**Bwa-mem**	**GraphMap**	**marginAlign**	**minimap2**
**Parameters**	–x ont2d	Default	Default	-ax map-ont	–x ont2d	Default	Default	-ax map-ont
**No. reads mapped (%)**	24,884 (8.32%)	23,652 (7.91%)	26,034 (8.71%)	21,574 (7.21%)	234,215 (78.3%)	226,696 (75.8%)	229,328 (76.7%)	219,611 (73.4%)
**No. genomes detected (%)**	66 (76.7%)	66 (76.7%)	67 (77.9%)	66 (76.7%)	26 (100%)	26 (100%)	26 (100%)	25 (96.2%)
**Base-level sensitivity [a]**	69.7%	70.6%	73.2%	68.9%	76.7%	76.7%	78.4%	75.5%
**Quantitative accuracy [b]**	0.910	0.915	0.889	0.895	0.810	0.807	0.801	0.631
**Depth of coverage (mean)**	109.1x	111.7x	110.9x	97.3x	10.8x	10.4x	10.5x	10.2x

Supplementary Table 2: Benchmarking of different read alignment tools for MinION long reads.
Metrics describing the alignment of MBARC-26 mock community DNA spiked with metagenome sequins (Mix A).
[a] Base-level sensitivity is defined as the fraction of reference bases covered by aligned reads.
[b] Quantitative accuracy is defined as the correlation coefficient (R^2) obtained after fitting a regression model to observed coverage plotted against input concentration.

Sample	No. contigs	No. contigs (> 500 nt)	Largest contig (nt)	Total length (nt)	N50 (nt)	Sequins assembled (%) [a]
**Natural soil (rep 1)**	597,805	3,329	62,662	2,787,866	801	47.25
**Natural soil (rep 2)**	806,207	3,012	12,320	2,732,930	932	58.49
**Natural soil (rep 3)**	794,158	9,707	13,920	10,954,025	1,266	41.06
**Natural rhizosphere (rep 1)**	749,186	3,748	50,462	7,499,443	3,659	55.86
**Natural rhizosphere (rep 2)**	435,627	298	12,907	374,725	1,444	51.18
**Natural rhizosphere (rep 3)**	1,892,185	2,647	67,112	2,991,243	1,247	68.45
**Natural root (rep 1)**	744,827	2,172	85,211	2,556,162	1,209	55.56
**Natural root (rep 2)**	1,327,735	9,289	61,300	13,057,171	1,835	61.99
**Natural root (rep 3)**	1,102,064	3,683	12,888	2,897,687	735	54.61
**Regenerated soil (rep 1)**	1,022,403	4,872	61,715	3,951,070	763	62.56
**Regenerated soil (rep 2)**	1,025,514	9,096	6,208	7,907,801	878	59.25
**Regenerated soil (rep 3)**	2,646,439	7,560	58,890	10,026,679	1,703	66.86
**Regenerated rhizosphere (rep 1)**	1,459,559	715	92,206	1,050,421	1,814	63.22
**Regenerated rhizosphere (rep 2)**	1,137,384	3,359	19,317	2,512,828	697	60.43
**Regenerated rhizosphere (rep 3)**	2,143,634	1,030	28,051	1,075,179	1,100	63.72
**Regenerated root (rep 1)**	986,819	2,103	209,468	2,912,602	1,661	54.52
**Regenerated root (rep 2)**	1,239,283	6,453	166,270	9,454,508	1,858	59.38
**Regenerated root (rep 3)**	981,887	4,720	195,076	4,913,643	1,029	52.76

Supplementary Table 3: Assembly statistics for saltmarsh metagenome samples.
[a] Refers to the fraction of total sequins reference genomes that are *de novo* assembled into contigs.
