# Supplementary material for: Synthetic microbe communities provide internal reference standards for metagenome sequencing and analysis
Source: Nat Commun. 2018 Aug 6;9:3096. doi: 10.1038/s41467-018-05555-0 (PMC6078961; doi:10.1038/s41467-018-05555-0)
Supplement: Supplementary file 3 — Description of Additional Supplementary Files [file 41467_2018_5555_MOESM3_ESM.pdf]

Description of Additional Supplementary Files

File Name: Supplementary Data 1
Description: Supplementary Data 1 is a table containing details of all 86 synthetic DNA standards ('sequins') that comprise the artificial microbial community developed in this study. Columns B – G provide information on each metagenome sequin, including its identification number (ID), length (bp), GC content (%), DNA sequence (5' – 3'), and its relative abundance in Mixes A and B (respectively). Columns H – M provide information on the source microbe from which each respective sequin was derived (where applicable), including its name, accession ID (RefSeq), genome size (bp), GC content (%), number of 16S rRNA copies, and its isolation source (respectively). Note that the first 16 sequins listed were designed as randomly generated sequences, thus have no source microbe.
